# Methylation-Based Classification of Cervical Squamous Cell Carcinoma into Two New Subclasses Differing in Immune-Related Gene Expression

**DOI:** 10.3390/ijms19113607

**Published:** 2018-11-15

**Authors:** Xia Li, Yunpeng Cai

**Affiliations:** Research Center for Biomedical Information Technology, Shenzhen Institutes of Advanced Technology, Chinese Academy of Sciences, 1068 Xueyuan Avenue, Shenzhen University Town, Shenzhen 518055, China

**Keywords:** methylation, cervical cancer, classification, immune, neo-epitopes

## Abstract

Cervical cancer is traditionally classified into two major histological subtypes, cervical squamous cell carcinoma (CSCC) and cervical adenocarcinoma (CA). However, heterogeneity exists among patients, comprising possible subpopulations with distinct molecular profiles. We applied consensus clustering to 307 methylation samples with cervical cancer from The Cancer Genome Atlas (TCGA). Fisher’s exact test was used to perform transcription factors (TFs) and genomic region enrichment. Gene expression profiles were downloaded from TCGA to assess expression differences. Immune cell fraction was calculated to quantify the immune cells infiltration. Putative neo-epitopes were predicted from somatic mutations. Three subclasses were identified: Class 1 correlating with the CA subtype and Classes 2 and 3 dividing the CSCC subtype into two subclasses. We found the hypomethylated probes in Class 3 exhibited strong enrichment in promoter region as compared with Class 2. Five TFs significantly enriched in the hypomethylated promoters and their highly expressed target genes in Class 3 functionally involved in the immune pathway. Gene function analysis revealed that immune-related genes were significantly increased in Class 3, and a higher level of immune cell infiltration was estimated. High expression of 24 immune genes exhibited a better overall survival and correlated with neo-epitope burden. Additionally, we found only two immune-related driver genes, *CARD11* and *JAK3*, to be significantly increased in Class 3. Our analyses provide a classification of the largest CSCC subtype into two new subclasses, revealing they harbored differences in immune-related gene expression.

## 1. Introduction

Cervical cancer is the second most prevalent cancer in females worldwide and can be traditionally classified into two common histological subtypes: cervical squamous cell carcinoma (CSCC) and cervical adenocarcinoma (CA). Among these, CSCC accounts for approximately 80% of all cervical cancer cases [1], with most of the remaining cases being CA. Evidences have shown that differences exist between these two subtypes, including risk factors [2], incidence rates [3], clinical features [4], and mutations [5]. The treatment adopted for these two subtypes is typically similar [6], while previous studies have reported relatively different outcomes [1].

In addition to the histological difference, cervical cancer also shows heterogeneity related to microenvironments such as hypoxia, variation in response to treatment, risk of metastasis, and gene expression [7,8,9,10]. Intratumoral metabolic and gene mutation heterogeneities are also observed in cervical cancer [11,12]. It is possible that the subpopulations present will have distinct molecular profiles with different levels of intrinsic response to therapy. Accurate subclass identification and characterization of the underlying mechanism are pivotal for our understanding of the disease and for the guiding of personalized therapies.

With the emergence of genome wide profiling techniques, large genomic datasets have become available for the discovery of new cancer subclasses. DNA methylation arrays measure the methylation status of thousands of CpG sites (or CG nucleotide) across the genome [6], and can also be used for cancer classification. Increasing evidence has shown that methylation profiling may reveal additional complexity that is not captured at the expression level or through genetic profiling [13,14,15], being able to delineate biologically relevant tumor subgroups [13,16,17]. In cervical cancer, changes in DNA methylation have been reported to play a critical role during cervical tumorigenesis [18,19,20]. In particular, hypermethylation of promoters is associated with the silencing of tumor suppressor genes, such as apoptosis-related genes and those involved in the cell cycle, DNA repair, and WNT (Wnt is an acronym in the field of genetics that stands for ‘Wingless/Integrated’) pathways [18]. The changes in methylation may result in aberrant gene expression, which consequently modifies the biological characteristics of the cancer. This prompted us to assess whether the DNA methylation profiles could divide cervical cancer patients into any new subgroups.

The large cervical cancer dataset generated by high-throughput genomic technologies and provided by The Cancer Genome Atlas (TCGA) offers a rich resource and a new opportunity to decipher the biological variability of tumors. In the present study, we performed clustering analysis using 307 cervical cancer patients’ DNA methylation data from TCGA. By combining the clustering results with the clinical information, we found that the largest cervical cancer subtype CSCC could be divided into two new subclasses, while the CA subtype stayed a single separate subclass. Based on this classification, we moved further to a detailed characterization of their differences by integrating gene expression analysis. We showed that these three subclasses displayed intrinsic differences in the methylation level and gene expression. Interestingly, we observed that for the CSCC subclasses the hypomethylation differences mainly occurred in the promoter regions. In one CSCC subclass, five transcription factors (TFs) showed enrichment in lowly methylated promoters and 28 of their highly expressed target genes were functionally enriched in the immune pathway. What’s more, gene function analysis revealed the differentially expressed genes between the two CSCC subclasses to be enriched in several immune response pathways. High expression of 24 genes involved in these pathways exhibited a better overall survival and correlated with predicted neo-epitope burdens. Finally, when assessing the differentially expressed TFs and driver genes, we noticed that only two immune-related driver genes were differentially expressed between the two CSCC subclasses.

## 2. Results

### 2.1. Analysis of DNA Methylation Identifies Two New Subclasses of CSCC

**Figure 1:**
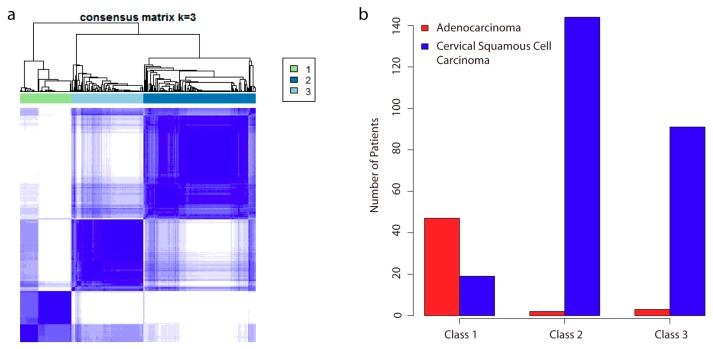
Identification of subclasses and association with histological status. (**a**) Consensus clustering of DNA methylation reveals three distinct subclasses of cervical cancer. K is the number of clusters generated. The heat map is a visual representation of the consensus matrix, which is a matrix of sample pairs. Each matrix entry measures the proportion of times the pair’s samples are clustered together across resampling iterations. In the heat map, values ranging from 0 (corresponding to two samples that are never clustered together) to 1 (corresponding to two samples that are always clustered together) are represented by white to dark blue, respectively. Samples in the matrix are ordered according to their cluster, resulting in a block-diagonal matrix. A dendrogram atop the heatmap is shown, which represents each cluster 1, 2, and 3, respectively; (**b**) bar plots show the number of patients in each subclass and their histological status.

To identify subgroups of samples, we performed unsupervised hierarchical clustering based on DNA methylation data. We selected the top 30,000 most variable probes that showed the highest median absolute deviation (MAD) across beta values for clustering (Figure S1a). Alternatively, the top 20,000 and 40,000 probes were also chosen for clustering, respectively. We observed the clustering based on the top 30,000 and 40,000 probes generated similar results, and the separation of patients was more distinct than the clustering based on the top 20,000 probes (Figure S1b). In addition, we also chose the probes located in the promoter regions for clustering. As we observed the promoter probes showed relatively lower MAD (Figure S1a), the top 10,000, 20,000, and 30,000 most variable promoter probes were used. Similarly, we noticed that the clustering results with the top 20,000 and 30,000 promoter probes performed better than the one with top 10,000 promoter probes, but achieved similar clustering structures (Figure S1b). By comparing the clusters generated from all probes versus promoter probes, we found that both results achieved three major clusters consistently. However, we noticed that there existed three subgroups in Cluster 1 that failed to be divided by promoter-probe clustering, while they could be identified by all-probe clustering (Figure S1b). Taken these results, we considered the clustering based on the top 30,000 probes, and used the three distinct clusters for subgroup separation. Using the clinical information, surprisingly, we found the derived clusters exhibited strong association with histological status (Figure 1). The patients assigned to Class 1 were mainly those with the CA subtype. Interestingly, Class 2 and Class 3 divided the CSCC subtype into two separate subclasses. The clusters derived from promoter probes also showed similar histological associations (Figure S1b). We calculated the mean beta values for all the probes in each subclass and noticed Class 1 showed a higher methylation level (Student’s *t*-test, *p*-value < 0.001; Figure S2). Although in the same CSCC subtype, Class 3 showed a lower global methylation level as compared with Class 2 (Student’s *t*-test, *p*-value < 0.001; Figure S2).

### 2.2. Lowly Methylated Promoters in Class 3 Show Enrichment in the Binding of 5 TFs

**Figure 2:**
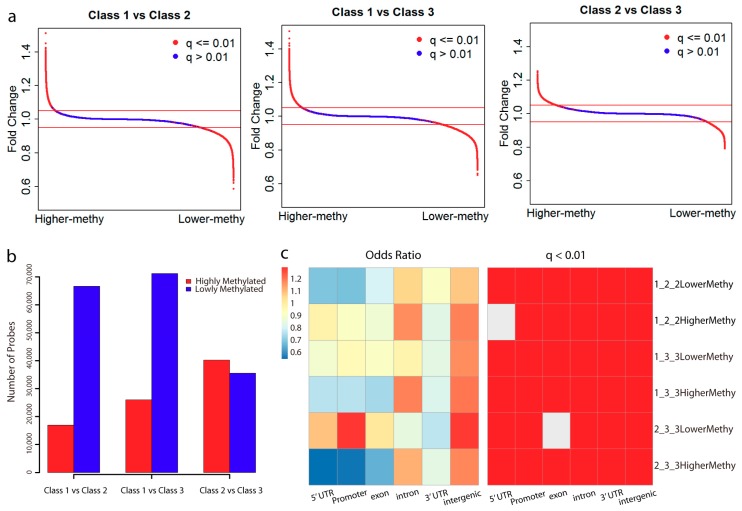
Differentially methylated probes and their genomic distribution. (**a**) Distribution of probes according to their fold change between each two subclasses. Probes with a *q*-value less than 0.01 are marked in red and the remaining in blue. Horizontal lines in red represent fold change = 1.05 and fold change = 0.95, respectively. Probes with a fold change > 1.05 and a *q*-value < 0.01 were selected as highly methylated, and a fold change < 0.95 and a *q*-value < 0.01 as lowly methylated. (**b**) Distribution of the number of highly and lowly methylated probes between each of the subclasses. (**c**) The genomic region enrichment of differentially methylated probes between subclasses. We calculated the number of differentially methylated probes, and the number of all probes on the bead array located in each genomic region. Fisher’s exact test was used to test the enrichment. Heat maps show the odds ratio and adjusted *p*-value for each category. “1_2_2LowerMethy” represents the lowly methylated probes in Class 2 in comparison with Class 1, “1_2_2HigherMethy” represents the highly methylated probes in Class 2 in comparison with Class 1, and so on.

Based on the methylome of each subclass, we then analyzed the differentially methylated probes between each of them (Figure 2a). In contrast to Class 1, we observed there were more lowly methylated probes in Class 2 and Class 3 (Figure 2b). This is consistent with the global methylation difference in which Class 1 showed a higher methylation level. However, there were more differentially highly methylated probes in Class 3 as compared with Class 2 (Figure 2b).

By taking all the probes in the Infinium HumanMethylation 450 BeadChip array from TCGA methylation profiles, we performed genomic enrichment analysis for the differentially methylated probes (Figure 2c). Interestingly, the differentially methylated probes between CSCC and CA type mainly occurred in the intergenic and intron regions. Notably, even in the same CSCC type, we found the differentially methylated probes between Class 2 and Class 3 displayed opposite enrichment. The highly methylated probes in Class 3 mainly enriched in the intergenic and intron regions. However, the lowly methylated probes displayed strong enrichment in the promoter region (Odds ratio: 1.299963; *q*-value: 1.79 × 10^−124^), followed by the intergenic and 5′ untranslated regions (UTR). Thus, different from other methylation changes that occurred in the intergenic and intron regions, the hypomethylation of the promoter and 5′ UTR regions may contribute to the separation of Class 3 from Class 2. In addition, these observations can also explain why we failed to identify some subgroups with promoter-probe clustering, since the methylation changes on the majority of probes mainly occurred in the intergenic and intron regions

**Figure 3:**
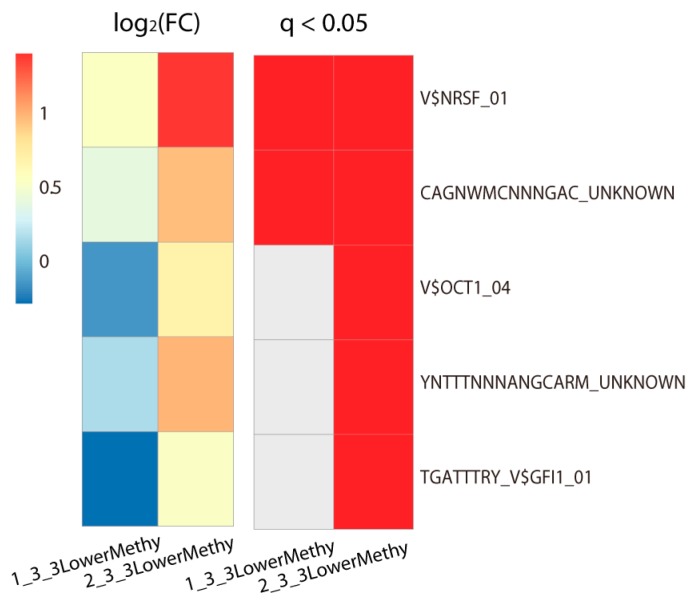
TFs binding enrichment around the lowly methylated promoters in Class 3. We found only these lowly methylated promoters in Class 3 showed significant TFs binding enrichment (Fisher’s exact test, adjusted *p*-value < 0.05). Heat maps show the log2 fold enrichment and significance of each TF binding around these promoters. The high value of the log2 fold enrichment means the TF is highly enriched. The significance of enrichment is marked in red (*q*-value less than 0.05) in the right heat map. All TFs in the right side of the heat map were obtained from the Molecular Signatures Database (MSigDB). “1_3_3LowerMethy” represents the lowly methylated probes in Class 3 in comparison with Class 1; “2_3_3LowerMethy” represents the lowly methylated probes in Class 3 in comparison with Class 2. FC, fold change (see Methods).

As the methylation of gene’s promoter is usually linked to gene expression [21], we extracted probes located in the promoter region from all differentially methylated probes, and subsequently obtained their regulated target genes. On the other hand, the methylation level of promoters can affect TFs’ binding, which could also regulate gene expression. Thus we then evaluated which TFs could bind to the promoter region of these genes. Among the target gene sets of those differentially highly or lowly methylated promoters in each subclass, only the lowly methylated promoters in Class 3 showed significant enrichment in TF binding (Fisher’s exact test, adjusted *p*-value < 0.05; Figure 3). This indicated that these TFs’ binding promoter regions were lowly methylated in Class 3. In comparison with Class 1, there were only two TFs that were significantly enriched, *NRSF* (Neuron-Restrictive Silencer Factor) (Fisher’s exact test, adjusted *p*-value = 2.9 × 10^−4^) and one unknown TF (Fisher’s exact test, adjusted *p*-value = 0.023). In contrast, five TFs showed enrichment in the lowly methylated promoter regions in Class 3 as compared with Class 2, especially *NRSF* (Fisher’s exact test, adjusted *p*-value = 1.9 × 10^−9^) and *OCT1* (Fisher’s exact test, adjusted *p*-value = 7.99 × 10^−4^). Another unknown TF (Fisher’s exact test, adjusted *p*-value = 0.0084) also significantly enriched.

### 2.3. Analysis of Differentially Expressed Genes between Subclasses and Their Correlation with Methylation

**Figure 4:**
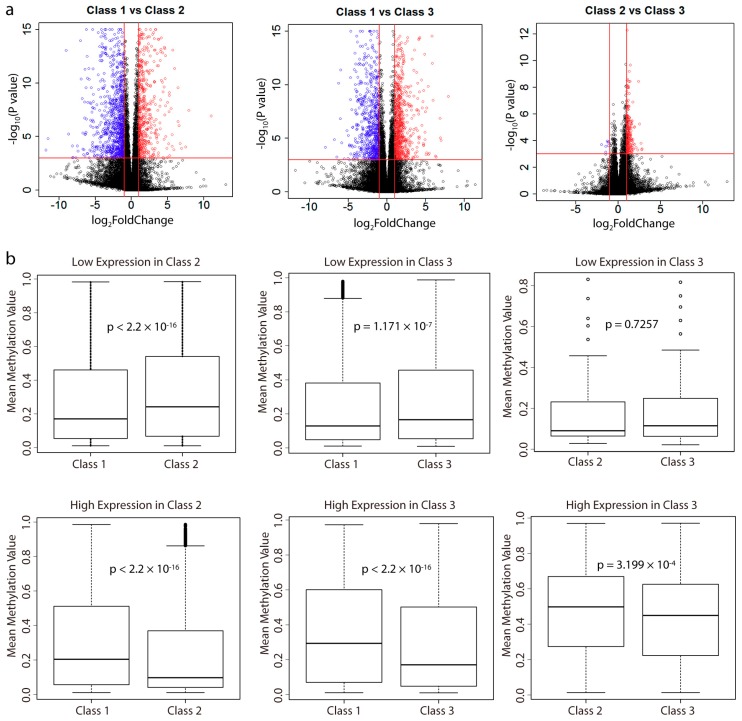
Differentially expressed genes and correlation with methylation. (**a**) Significance of gene expression differences between each subclass. Each dot represents one gene. The x axis shows the gene expression difference by a log2 transformed fold change while the y axis shows significance by a −log_10_ transformed *p*-value. Vertical lines in red represent log_2_FoldChange = −1 and log_2_FoldChange = 1, respectively. Horizontal line in red represents *p*-value = 0.001. We defined differentially expressed genes if their absolute values of log_2_FoldChange larger than 1 and *p*-value less than 0.001 (dot in red means high expression and blue means low expression). (**b**) Boxplot shows the distribution of the mean beta values of differentially expressed gene sets’ promoters probes. The small black circle represents outlier. The black rod from top to bottom represents the maximum, upper quartile, median, lower quartile, and minimum value, respectively. The black dotted line represents the values between the intevals. *p*-value was calculated using a two-sided Student’s *t*-test.

Using the RNA-seq data, we performed differential gene expression analysis between the subclasses (Figure 4a). In total, there were more lowly expressed genes in Class 2 as compared with Class 1, and more highly expressed genes in Class 3 as compared with Class 2 (Figure S3a). Class 3 showed a comparable number of differentially expressed genes with Class 1. However, as we have shown above, Class 2 harbored more lowly methylated probes as compared with Class1, while Class 3 was more highly methylated than Class 2, which is inconsistent with the number of differentially expressed genes between them. By examining the overlap of genes between these differentially methylated genes and differentially expressed genes, we observed that the majority of differentially methylated genes did not lead to differential gene expression (Figure S3b–d). However, the differentially expressed genes did show a correlation with the differentially methylated genes. As we observed, there were more lowly methylated genes with high expression, and more highly methylated genes with low expression.

For these differentially expressed genes, we calculated the mean beta values of their promoter probes and the results showed that the gene expression of these differentially expressed genes was negatively correlated with the methylation level of their promoters (Figure 4b). Thus, these results indicated that the differential gene expression changes were regulated by the methylation levels of their promoters.

### 2.4. Immune-Related Genes are Highly Expressed in Class 3

**Figure 5:**
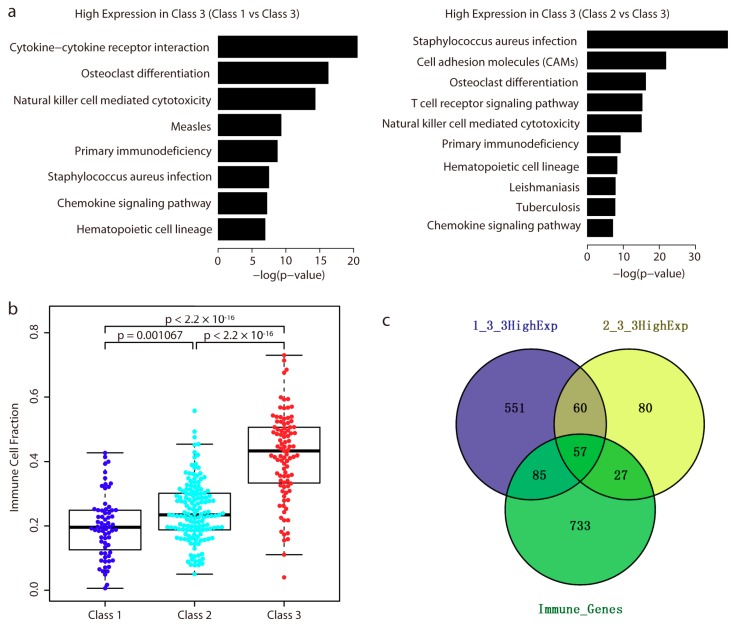
Highly expression of immune related genes and high level of immune cell fraction in Class 3. (**a**) Bar plots show the significantly enriched pathways of the highly expressed genes in Class 3. *p*-value was obtained from DAVID analysis. Logarithmic transformation (base 10) was applied to the *p*-value. The significantly enriched pathway was considered if the *p*-value less than 0.001. (**b**) Distribution of immune cell fraction in three groups. Boxplot shows high immune cell fraction in Class 3. The subgroup was colored in blue (Class 1), cyan (Class 2), and red (Class 3), respectively. The difference in the immune cell fraction between different group was performed by two-sided Student’s *t*-test. (**c**) Venn representation of overlaps among highly expressed genes in Class 3 as compared with Class 1 (“1_3_3HighExp”), highly expressed genes in Class 3 as compared with Class 2 (“2_3_3HighExp”), and all genes involved in immune pathways (“Immune_Genes”) in (**a**).

For each set of differentially expressed genes between each subclass, gene function analysis using Database for Annotation, Visualization, and Integrated Discovery (DAVID) [22] showed that only those highly expressed genes in Class 3 were significantly enriched in the immune response system (*q*-value < 0.001; Figure 5a). Based on each class’s methylation profiles, we then quantified the level of immune cells infiltration by calculating the immune cell fraction in each sample by EpiDISH (https://bioconductor.org/packages/release/bioc/html/EpiDISH.html) [23]. As shown in Figure 5b, the immune cell fraction in patients of Class 3 ranked the highest of all three groups, followed by that of Class 2 and Class 1. We also used the beta values of the top 30,000 most variable probes, and the differentially methylated probes between each subclass to calculate the immune cell fraction, and the results remained the same (Figure S4).

We above showed that five TFs were significantly enriched in the lowly methylated promoter regions in Class 3. Among those TFs’ target genes, 28 were highly expressed in Class 3 (Figure S5). In addition, we listed the expression of them in normal cervix tissue and cancer tissue (Table S1). We even observed their high expression in Class 3; some of them were also highly expressed in cervix tissue. Gene function analysis showed they were significantly enriched in the immune pathway (category: Kyoto Encyclopedia of Genes and Genomes (KEGG) PATHWAY, term: T cell receptor signaling pathway, *q*-value: 0.035; Fisher’s exact test, *p* = 2.1 × 10^−5^). This suggested that the hypomethylation of the promoters of these genes might recruit the five TFs’ binding, leading to increased expression of the 28 target genes.

Of those highly expressed genes in Class 3, a total of 117 genes were highly expressed as compared with both Class 1 and Class 2. Notably, among them, 57 genes were involved in the immune system (Figure 5c). This gene list represented one immune-related gene signature with high expression in Class 3. We next asked whether these genes had any prognostic relevance. Interestingly, of these 57 genes, survival analysis showed that patients with high expression of 24 genes exhibited a better overall survival (Table 1). The proteins encoded by *CD3E*, *CD247*, and *CD3D* are components of the T cell receptor. Four chemokine receptors (*CCR5*, *CXCR3*, and *CCR2*), one interleukin-10 family (*IL10RA*), one interleukin-1 family (*IL18RAP*), and one chemokine (*CXCL9*) are involved in the cytokine–cytokine receptor interaction. In addition, we also checked the clinical relevance of the 24 genes from the Human Protein Atlas [24], and found high expression of them was also reported to exhibit better survival. Notably, four genes (*CD3D*, *CD3E*, *CD7*, and *SELL*) were reported to be associated with favorable prognostic value (Table 1).

In addition, we observed that a total of 84 immune-related genes were highly expressed in Class 3 as compared with Class 2 (no immune genes were lowly expressed between these two classes) (Figure 5c). Thus we made a clinical comparison of the patients in CSCC based on the 84 genes’ expression. Two groups were divided, and survival analysis showed the group with high expression of the 84 genes displayed better overall survival (logrank (Mantel–Cox) test, *p* = 0.01, Figure S6).

### 2.5. Correlation of 24 Immune-Related Genes’ Expression with Predicted Neo-Epitope Burden

We above showed the 24 immune-related genes displayed potential prognostic characteristics since their high expression was associated with better survival. This indicates patients with a stronger immune system are more likely to live longer. On the other hand, the neo-epitopes, which could be derived from mutations in patients, if presented on major histocompatibility complex class I molecules (MHC-I), may render the tumor more susceptible to the immune system as they would be recognized as “nonself” neo-antigens. In our recent work, we demonstrated that a higher number of neo-epitopes in these same cervical cancers exhibited an association with better survival [25]. Using those predicted neo-epitopes from our previous work, we therefore investigated whether higher expression levels of the 24 genes were correlated with the number of neo-epitopes. Spearman correlation was calculated to assess the association of the 24 genes’ expression with the number of neo-epitopes across patients. With the exception of *ITK*, *PIK3CG*, *SELL*, and *BTK*, we detected a significant positive correlation between the rest genes’ expression and neo-epitope burden (Table 2). For comparison, we also randomly selected 24 genes across the genome to assess the association of their expression with the number of neo-epitopes. Furthermore, we repeated the random gene selection and the correlation significance calculation for 1000 times. Among each of the 1000 times’ computing, we compared the difference in *p*-values between the 24 prognostic genes’ and randomly selected genes’, and calculated the false discovery rate. A false discovery rate of 0.005 indicated the significant correlation between high expression of those prognostic genes and the neo-epitope burden did not occur by chance. We also calculated the number of Spearman’s rho among the 24 genes higher than the 24 randomly selected genes’ in each sampling. It turned out that the majority of the 24 genes had a higher rho score against the random genes in the random sampling (Figure S7).

### 2.6. Differentially Expressed TFs and Driver Genes between Each Subclass

**Figure 6:**
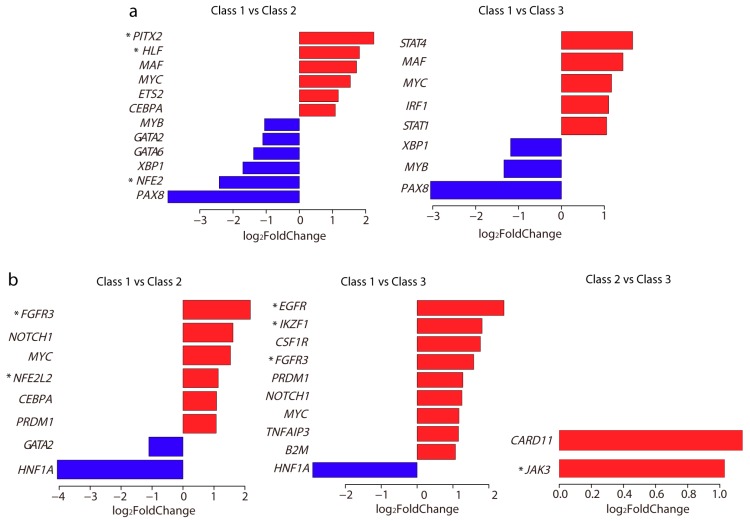
Significantly expressed TFs and driver genes. Bar plots of log_2_ fold change in differentially expressed TFs (**a**) and driver genes (**b**) between each subclass. The bar in red means high expression and blue means low expression. Gene marked with an asterisk indicates that its high or low expression was associated with patient survival (Figure S8).

In addition to the roles of TFs in the regulation of gene expression through their binding (or not) around promoter regions, the high or low expression of TFs can also influence gene expression. Thus we examined whether any TFs were differentially expressed between each subclass. Surprisingly, a total of 15 TFs were significantly differentially expressed in Class 2 and Class 3 as compared with Class 1 (Figure 6a). However, none of them were found to show differential expression between Class 2 and Class 3. Among them, the low expression of *NFE2* and *PITX2*, and high expression of *HLF* on patients displayed better survival (Figure S8).

Driver genes play important roles in cancer development. We next examined whether these three subclasses showed significant differential gene expression in driver genes. We obtained 138 driver genes from a previous report [26], and found a total of 15 driver genes were significantly differentially expressed between each subclass (Figure 6b). Among them, only two driver genes *CARD11* and *JAK3* showed significant differential expression between Class 2 and Class 3, both with high expression in Class 3. Interestingly, these two genes were also related to the immune response system. *CARD11* is involved in both the T cell and B cell receptor signaling pathways. *JAK3* is commonly expressed on T cells [27] and is also involved in the JAK (Janus kinases)/STAT (Signal Transducer and Activator of Transcription proteins) signaling pathway. Survival analysis showed that high expression of *IKZF1*, *FGFR3*, *NFE2L2*, and *JAK3*, while low expression of *EGFR*, exhibited better survival (Figure S8).

In addition, we extracted the promoter probes for these differentially expressed TFs and driver genes and calculated the mean bata values in each subclass (Figure S9). In general, the average methylation level in these genes’ promoters was also negatively correlated with their gene expression.

## 3. Discussion

In this study, we applied unsupervised analysis to cervical cancer samples using methylation profiles to reveal new subclasses that correlated with histological status. Specifically, we revealed two subpopulations existing in the CSCC subtype. In addition to the difference in methylation level, these two subclasses also showed differences in TFs binding around the promoter regions and in gene expression. Gene function assessment revealed the two subclasses harbored major differences in the immune-related gene expression. The differences in the methylation level, together with the TFs binding around the promoters, might play roles in inducing and maintaining the different phenotypes. Our findings suggest high interpatient heterogeneity in cervical cancer, and are useful for cervical cancer classification and prediction of prognosis.

Additionally, integrative clustering based on multiple omics data could also be applied to decipher subpopulations among patients [28]. One recent study of 228 cervical cancers from TCGA integrated various data types, including copy number, DNA methylation, mRNA, and microRNA data, and also revealed the molecular heterogeneity of cervival carcinomas [29]. Interestingly, they also identified three clusters: two squamous clusters and an adenocarcinoma-rich cluster, which agreed with our findings. However, we obtained the histological associations directly from the clustering based on single methylation data. Unlike the immune gene expression differences between the two CSCC characterized in our study, they showed the two squamous clusters differed in the expression of keratin gene family members. This inconsistence might be due to the different sample size used. Moreover, we also observed the high immune gene expression CSCC subclass displayed high level of immune cell estimate, which further supported the existence of immune subtype in CSCC. They also performed unsupervised hierarchical clustering based on single DNA methylation data. Also, three clusters were identified: a small ‘CpG island hypermethylated’ (CpG island methylator phenotype (CIMP)-high) cluster, a CIMP-intermediate cluster and a CIMP-low cluster. By comparing with the integrative clusters, they found most of the patients in the adenocarcinoma cluster were CIMP-high, whereas the two squamous clusters contained a mixture of CIMP-intermediate and CIMP-low patients. In our study, we also showed the CA subclass Class 1 displayed a higher methylation level and harbored more differentially hypermethylated probes as compared with the two CSCC subclasses. Again, these results were also consistent. Furthermore, we revealed the hypomethylation of the promoter and 5′ UTR regions may contribute to the separation of the two CSCC subclasses. Thus, even based on different sample size and clustering approaches, our study agreed with the main findings of the TCGA paper. However, our study provided more detailed molecular characterizations that have not been extensively explored in cervical cancer.

Some types of human papilloma virus (HPV) infection, especially HPV 16 and HPV 18, present the greatest risk factor for cervical cancer. HPV infection has been reported to be associated with the regulation of DNA methylation. For instance, the HPV 16 E7 oncoprotein is associated both in vitro and in vivo with the DNA methyltransferase *DNMT1* and stimulates its enzymatic activity [30]. On the other hand, it has been suggested that HPV infection could alter the immune response in the pathogenesis of cervical cancer [31]. In the present study, we observed the methylation profiles divided the CSCC subtype into two separate subclasses that were different in immune-related gene expression. Among those immune response pathways, we observed the cytokine–cytokine receptor interaction pathway was highly expressed in Class 3. Interestingly, previous studies have shown that the HPV E6 and E7 proteins can directly interact with cytokines that are induced following infection [32,33,34]. Consequently, this results in the blockade of apoptosis and the continued acquirement of proliferation ability. In addition, we noticed *STAT1* was highly expressed in Class 3. It is a key TF that regulates the interferon response which is also activated following viral infection. However, it has been reported that this activity could also be inhibited by HPV proteins [17]. It appears that HPV infection may induce the immune system response, but on the other hand, those HPV oncoproteins may act at several levels to interfere with this response. In this study, we only observed 15 patients had HPV infection data. Due to the lack of enough information in the TCGA regarding HPV infection, we were unable to examine the link between the subclasses and HPV infection status. Further detailed investigation into the molecular mechanisms is warranted.

Among those significantly expressed TFs and driver genes, we observed that the expression levels of eight genes were associated with patient survival. An early report suggested that *NFE2* could play a role in megakaryocyte transformation [35], and that knockdown of *NFE2*-related factor 2 (*NRF2*) in cervical cancer could enhance the efficacy of anticancer drugs [36]. This suggests the ability of *NFE2* to promote tumorigenesis. Here we showed that the low expression of *NFE2* was associated with better survival, which revealed its similar role in cervical cancer. Furthermore, it was particularly significantly lowly expressed in Class 2 and highly expressed in Class 1. Increased expression of *PITX2* has a critical function in ovarian cancer progression [37], while in our data, we observed it was highly expressed in Class 2 and lowly expressed in Class 1. Previous study demonstrated *PITX2* serves as one promising predictive biomarker in esophageal squamous cell carcinoma prognosis [38]. We observed that low expression of *PITX2* was associated with better survival, which also displayed its prognostic characteristic in cervical cancer. *HLF* is one hypoxia response regulator, and its transcriptional role varies among tumor types [39]. In our data, it was highly expressed in Class 2 and lowly expressed in Class 1, and its high expression displayed better survival. *IKZF1*, one critical regulator of lymphocyte development [40], was highly expressed in Class 3 while lowly expressed in Class 1. This is also one immune related gene and its high expression was also associated with better survival. Our observation that the high expression of *EGFR* predicted poor survival in cervical cancer has been confirmed in a previous report [41]. More precisely, we reported *EGFR* was extremely highly expressed in the CSCC subclass Class 3 and lowly expressed in the CA subclass Class 1. The association of the high expression of *FGFR3* with better survival in cervical cancer was also confirmed by a recent study [42]. Here, we observed its high expression in the CSCC subtype (both Class 2 and Class 3) and also its association with better survival. *NFE2L2*, previously identified as a recurrently mutated gene in cervical cancer [31], was highly expressed in Class 2 and lowly expressed in Class 1. Also, its high expression was associated with better survival in our data. *JAK3*, one of two significantly highly expressed driver genes between the two CSCC subclasses, displayed better survival.

In addition to those eight genes displaying prognostic characteristics, we also identified 24 immune-related genes in Class 3 and their high expression was associated with better survival. Consistently, among these, a previous study using squamous cell cervical cancer samples demonstrated that the high expression of the T cell receptor component, *CD3E*, is correlated with improved patient survival [43]. From the Human Protein Atlas, it was shown that the high expression of *CD3E* was also associated with patients’ long-term survival in other types of cancer, such as endometrial cancer, melanoma, head and neck cancer, and breast cancer. For other immune-related genes, it will be interesting to investigate their involvement in other cancer types. It should be noted that we divided patients into high/low expression group based on the fourth/second quantile value. Thus other kind of group division method should give different clinical significance. In this study, we also checked these genes’ clinical relevance from the Human Protein Atlas where gene expression values from the 20th to 80th percentiles were used to group the patients. Consistently, the prognostic value of these genes remained the same as our examination. Specially, four genes (*CD3D*, *CD3E*, *CD7*, and *SELL*) were reported to be associated with favorable prognostic value in the Human Protein Atlas. It was noteworthy that a larger patient sample size should make the prognostic gene list more stable. For the immune-related genes, when the sample size is increased, there may be more genes included in the list, since other immune pathways would be identified as significantly enriched. More cervical cancer genomic data are expected in the future; thus we can stabilize the gene list, and a follow-up study for validation will also be feasible. Confirmation of those prognostic genes could represent biomarker signatures for each subclass, which will be helpful for large-scale classification and improvements in prognosis prediction. Nevertheless, in this study, we were still able to achieve reliable gene lists, paving the way for future exploration.

## 4. Materials and Methods

### 4.1. Summary of Samples

We downloaded methylation data for a total of 307 cervical cancer samples (Illumina Infinium Human DNA Methylation 450 platform, beta values) and clinical information for all patients from TCGA under Genomic Data Commons (GDC) (Bethesda, MD, USA). In total, 485,577 methylation probes were used to explore the DNA methylation profile on the genome scale. Beta values that ranging between 0 and 1 were used to represent the relative methylation level, which were measured as the ratio of the methylated probe intensity over all methylation probe intensities.

### 4.2. Consensus Clustering

We performed consensus clustering using the ConsensusClusterPlus [44] R package (R Core Team, Vienna, Austria). The top 30,000 most variable probes that showed the highest MAD across the beta values were selected for clustering. Alternatively, the top 20,000 and 40,000 probes were also chosen for clustering, respectively. In addition, we defined the promoter probe if the probe located in the region of 3000 bp around the transcription start site with 1500 bp upstream and 1500 bp downstream. The top 10,000, 20,000, and 30,000 most variable promoter probes were also used to perform hierarchical clusterings. The following settings were used in the consensus clustering: Number of resamplings: 1000; pItem = 0.9 (resampling frequency samples); pFeature = 0.9 (resampling frequency); Pearson distance metric; Ward linkage clustering method. We analyzed consensus matrices for the number of clusters k from 2 to 6 and found the most robust result with a 3-cluster solution.

Based on the methylome of each subclass, we calculated the mean beta values for all probes in each subclass. The two-sided Student’s *t*-test was applied to compare the global methylation difference between each subclass. A *p*-value less than 0.001 was considered to indicate significance.

### 4.3. Differential Methylation Analysis

We performed differentially methylation analysis for each probe based on the beta value [45] using the Samr [46] package in the R software (R Core Team, Vienna, Austria). The significance of differentially methylated probes between each subclass was performed. Probes with a fold change > 1.05 and a *q*-value < 0.01 were selected as highly methylated ones, and those with a fold change < 0.95 and a *q*-value < 0.01 as lowly methylated.

### 4.4. Genomic Region Enrichment

The genomic region, including intergenetic regions, the 5′UTR, whole exon regions, whole intron regions, and 3′ UTR were obtained from the University of California, Santa Cruz (UCSC) genome browser [47]. The promoter region was defined as 3000 bp around the transcription start site with 1500 bp upstream and 1500 bp downstream. The probe was taken as being located in each genomic region when its location overlapped with the corresponding region. We calculated the number of differentially methylated probes located in each genomic region. All the probes in the Infinium HumanMethylation 450 BeadChip array from TCGA methylation profiles were extracted, and the number of these probes located in each genomic region was also calculated. Fisher’s exact test was used to test the enrichment. Odds ratio and *p*-value were obtained, and the *p*-value was adjusted using the Benjamini and Hochberg method.

### 4.5. TFs Binding Enrichment and Target Gene Function

We extracted the differentially methylated promoter probes by checking whether those differentially methylated probes were located in the promoter regions. We then defined the highly or lowly methylated genes by way of evaluating whether their promoter contained differentially highly or lowly methylated probes (as described above). We excluded genes when their promoter harbored both highly and lowly methylated probes.

Thus, for comparison of each pair of subclasses, there were two sets of differentially methylated genes: highly methylated and lowly methylated genes. Next, we examined whether these differentially methylated genes’ promoters enriched in any TFs binding. We downloaded a total of 283 TFs’ target genes from MSigDB. For each set of differentially methylated genes *c*, we defined *N* as the total number of nonredundant target genes in MsigDB, *n_c_* as the number of differentially methylated genes, *K_s_* as the number of target genes for each TF *s*, and *k_sc_* as the number of differentially methylated genes that was found in each TF’s target genes. Fisher’s exact test was then performed to test whether each TF’s target genes were significantly enriched in those differentially methylated genes. The fold enrichment was defined as log_2_([(*k_sc_* + 1)/(*K_s_* + 1)]/[(*n_c_* + 1)/(*N* + 1)]), similar to one previous approach [48]. The adjusted *p*-value for each TF was calculated using the Benjamini and Hochberg method. The significantly enriched TFs binding was considered if the adjusted *p*-value less than 0.05.

For those significantly enriched TFs’ target genes with high expression in Class 3, we used DAVID [22] to perform gene function analysis. A *q*-value less than 0.05 was considered to indicate statistical significance.

### 4.6. Analysis of Differentially Expressed Genes and Gene Function

RNA sequencing raw reads count data were downloaded from IlluminaHiSeq_RNASeqV2 (Level 3) in TCGA under GDC. The DESeq [49] package in the R software (R Core Team, Vienna, Austria). was applied to the identification of differentially expressed genes between each subclass. The *p*-value was adjusted using the Benjamini and Hochberg method. We defined genes as differentially expressed when their absolute log_2_FoldChange was larger than 1 and the adjusted *p*-value was less than 0.001.

For each differentially expressed gene set in each subclass pair comparison, we extracted the probes of the promoter, and calculated the mean beta values of the probes in each subclass. A two-sided Student’s *t*-test was used to compare the differences in the methylation levels of these promoters between each pair of two subclasses. A *p*-value less than 0.001 was considered to indicate significance.

Gene function analysis of these differentially expressed genes was performed using DAVID. The significantly enriched pathway was considered if the *q*-value less than 0.001.

### 4.7. Immune Cell Fraction Calculation

Based on DNA methylation data, immune cell fraction was predicted by EpiDISH algorithm [23]. In general, the value represented the level of immune cells infiltration in tumor. We also performed immune cell fraction calculation based on the beta values of the top 30,000 most variable probes, and the differentially methylated probes between each subclass.

### 4.8. Correlation Analysis of Gene Expression with Predicted Neo-Epitope Burden

Using the exome-sequencing data of the same cervical cancer patients from TCGA, we have previously predicted the neo-epitopes based on somatic mutations [25]. In general, the neo-epitopes were obtained if the mutant peptides showed strong binding affinity with MHC-I. Based on these results, we calculated the number of neo-epitopes in each patient. The normalized read counts obtained from IlluminaHiSeq_RNASeqV2 (Level 3) in TCGA were taken as the gene expression values. For each of the 24 prognostic genes in our study, Spearman correlation was calculated to assess the association of gene expression with the number of neo-epitopes across patients. A two-tailed *p*-value of less than 0.05 was considered to indicate statistical significance. We randomly sampled the same number of genes from all the genes in the human genome without replacement. For each of those randomly selected genes, we also computed the association of gene expression with number of neo-epitopes. We performed 1000 such random samplings and calculations. In each sampling, we compared the *p*-values of those 24 prognostic genes and the randomly sampled genes using a two-sided Student’s *t*-test. If the mean of those 24 prognostic genes’ *p*-values was less than the mean of the random genes’, and the significance of difference satisfied a *p*-value less than 0.001, we considered the correlation of those 24 prognostic genes’ expression with the neo-epitope burdens was significant in that sampling. A false discovery rate was then calculated based on those 1000 times’ comparison.

### 4.9. Survival Analysis

Survival curves were generated using the Kaplan–Meier method. For each gene, a patient was classified as high expression when the expression value was above the fourth quantile, and low expression when below the second quantile. Differences were evaluated using the logrank (Mantel–Cox) test. Overall survival was calculated from the time of initial diagnosis to death or censored to the time at which the patient was last known to be alive. A *p*-value less than 0.1 was considered statistically significant. Hazard ratios and associated 95% confidence intervals were calculated with the use of the Cox proportional-hazards model. All tests were two-sided and all calculations were performed with the R Version 3.3.1 statistical software (R Core Team, Vienna, Austria).

## 5. Conclusions

In conclusion, the present study investigated the methylation data obtained from TCGA to revisit the classification of cervical cancer subtypes, and identified two new subclasses in the CSCC subtype. By an integrative analysis of gene expression data, our results revealed major differences in immune-related gene expression among these two subclasses. Our results provide important insight into interpatient heterogeneity among cervical cancer, which improves our ability to classify these tumors and contributes to prognostic and diagnostic use in clinics.

## Figures and Tables

**Table 1:** Association of the expression of immune-related genes with patient survival.

Gene	Event Status	Events No.	*p* Value	Adjusted *p* value ^1^	Hazard Ratio	95% Confidence Interval	*p* Value ^2^	Adjusted *p* Value ^3^
*CCR5*	High Expression	60	0.0268	0.0804	0.3279	0.116–0.925	0.00194	0.00418
	Low Expression	58						
*CXCL9*	High Expression	61	0.0892	0.0931	0.4483	0.1734–1.159	0.00686	0.0087
	Low Expression	58						
*CXCR3*	High Expression	59	0.0806	0.0912	0.4847	0.2114–1.1114	0.00305	0.0048
	Low Expression	59						
*IL10RA*	High Expression	59	0.0705	0.0912	0.4541	0.1893–1.0892	0.002	0.0042
	Low Expression	55						
*IL18RAP*	High Expression	61	0.0102	0.0612	0.3637	0.1629–0.8123	0.00627	0.0084
	Low Expression	58						
*ITGAX*	High Expression	61	0.063	0.0912	0.4837	0.2212–1.0573	0.0249	0.0249
	Low Expression	56						
*CCR2*	High Expression	59	0.0549	0.0912	0.3987	0.1509–1.0532	0.000628	0.0023
	Low Expression	57						
*DOCK2*	High Expression	61	0.078	0.0912	0.486	0.2143–1.1025	0.0184	0.0201
	Low Expression	55						
*WAS*	High Expression	60	0.0836	0.0912	0.5171	0.2417–1.1063	0.00209	0.0042
	Low Expression	57						
*BTK*	High Expression	61	0.0949	0.0949	0.4938	0.2123–1.149	0.00251	0.0046
	Low Expression	56						
*CD3D* ^4^	High Expression	60	0.0648	0.0912	0.4393	0.179–1.078	8.46 × 10^−5^	0.0016
	Low Expression	57						
*CD3E* ^4^	High Expression	60	0.009	0.0612	0.3262	0.1347–0.7899	1.62 × 10^−4^	0.0016
	Low Expression	57						
*CD7* ^4^	High Expression	60	0.0361	0.0912	0.4499	0.209–0.969	5.51 × 10^−4^	0.0023
	Low Expression	58						
*CD48*	High Expression	60	0.046	0.0912	0.4262	0.18–1	0.0017	0.0042
	Low Expression	57						
*CD247*	High Expression	60	0.0654	0.0912	0.5043	0.24–1.0593	0.00321	0.0048
	Low Expression	58						
*CTLA4*	High Expression	58	0.0696	0.0912	0.4556	0.191–1.0882	0.00913	0.011
	Low Expression	57						
*GZMB*	High Expression	58	0.0664	0.0912	0.4189	0.1609–1.091	0.0028	0.0048
	Low Expression	58						
*ITGAL*	High Expression	60	0.0216	0.0804	0.3823	0.1634–0.8945	0.00426	0.006
	Low Expression	57						
*ITK*	High Expression	59	0.0253	0.0804	0.4266	0.1978–0.92	6.57 × 10^−4^	0.0023
	Low Expression	57						
*KLRK1*	High Expression	60	0.00449	0.05388	0.3	0.125–0.7236	2.65 × 10^−4^	0.0016
	Low Expression	57						
*PIK3CG*	High Expression	60	0.0819	0.0912	0.46	0.1879–1.126	0.0201	0.021
	Low Expression	54						
*PTPRC*	High Expression	60	0.0175	0.0804	0.3662	0.1546–0.8681	0.0124	0.0142
	Low Expression	56						
*SELL* ^4^	High Expression	62	0.000698	0.0168	0.2366	0.096–0.5824	2.25 × 10^−4^	0.0016
	Low Expression	57						
*SPN*	High Expression	61	0.0508	0.0912	0.4424	0.1909–1.0256	0.00163	0.0042
	Low Expression	56						

^1^ The adjusted *p*-value was calculated using the Benjamini and Hochberg method. ^2^ The *p*-value was obtained from the Human Protein Atlas. ^3^ The adjusted *p*-value from the Human Protein Atlas. ^4^ The gene is marked as “Prognostic, favorable” in cervical cancer from the Human Protein Atlas.

**Table 2:** Association of 24 immune related genes’ expression with neo-epitope burden.

24 Prognostic Genes	Spearman’s Rho	*p*-Value
*CD247*	0.18011804	0.011746072222
*ITGAL*	0.23202640	0.001098899936
*ITGAX*	0.18054878	0.011543220339
*ITK*	0.11669343	0.104244914224
*PIK3CG*	0.10671102	0.137596629299
*SPN*	0.18914430	0.008091618099
*CD3D*	0.21162724	0.002978712632
*CD3E*	0.22309120	0.001719277379
*CD48*	0.17823018	0.012672607086
*IL10RA*	0.18267087	0.010588223639
*DOCK2*	0.16089662	0.024639133354
*CD7*	0.21193808	0.002935714814
*PTPRC*	0.17346131	0.015304390326
*GZMB*	0.26446319	0.000186953723
*CTLA4*	0.23369399	0.001008922888
*WAS*	0.17836121	0.012606283349
*SELL*	0.06245491	0.385736421301
*KLRK1*	0.21769835	0.002234159280
*IL18RAP*	0.15330837	0.032376073551
*CXCL9*	0.32242557	0.000004289406
*CXCR3*	0.24277477	0.000626961434
*CCR2*	0.17927831	0.012150572695
*CCR5*	0.24935640	0.000439124718
*BTK*	0.13588272	0.058212535746

## Supplementary Materials

Supplementary materials can be found at http://www.mdpi.com/1422-0067/19/11/3607/s1.

## Abbreviations

CSCC	cervical squamous cell carcinoma
CA	cervical adenocarcinoma
TCGA	The Cancer Genome Atlas
TFs	transcription factors
MAD	median absolute deviation
DAVID	Database for Annotation, Visualization and Integrated Discovery
MHC-I	the major histocompatibility complex class I molecules
HPV	human papilloma virus
GDC	Genomic Data Commons
UTR	untranslated region
MSigDB	Molecular Signatures Database
NRSF	Neuron-Restrictive Silencer Factor
KEGG	Kyoto Encyclopedia of Genes and Genomes
JAK	Janus kinases
STAT	Signal Transducer and Activator of Transcription proteins
CIMP	CpG island methylator phenotype
UCSC	the University of California, Santa Cruz

## References

[1] Irie T., Kigawa J., Minagawa Y., Itamochi H., Sato S., Akeshima R., Terakawa N. (2000). Prognosis and clinicopathological characteristics of Ib-IIb adenocarcinoma of the uterine cervix in patients who have had radical hysterectomy. Eur. J. Surg. Oncol..

[2] International Collaboration of Epidemiological Studies of Cervical Cancer (2007). Comparison of risk factors for invasive squamous cell carcinoma and adenocarcinoma of the cervix: Collaborative reanalysis of individual data on 8097 women with squamous cell carcinoma and 1374 women with adenocarcinoma from 12 epidemiological studies. Int. J. Cancer.

[3] Smith H.O., Tiffany M.F., Qualls C.R., Key C.R. (2000). The rising incidence of adenocarcinoma relative to squamous cell carcinoma of the uterine cervix in the United States—A 24-year population-based study. Gynecol. Oncol..

[4] Hopkins M.P., Morley G.W. (1991). A comparison of adenocarcinoma and squamous cell carcinoma of the cervix. Obstet. Gynecol..

[5] Wright A.A., Howitt B.E., Myers A.P., Dahlberg S.E., Palescandolo E., Van Hummelen P., MacConaill L.E., Shoni M., Wagle N., Jones R.T. (2013). Oncogenic mutations in cervical cancer: Genomic differences between adenocarcinomas and squamous cell carcinomas of the cervix. Cancer.

[6] Shimada M., Nishimura R., Nogawa T., Hatae M., Takehara K., Yamada H., Kurachi H., Yokoyama Y., Sugiyama T., Kigawa J. (2013). Comparison of the outcome between cervical adenocarcinoma and squamous cell carcinoma patients with adjuvant radiotherapy following radical surgery: SGSG/TGCU Intergroup Surveillance. Mol. Clin. Oncol..

[7] Hockel M., Schlenger K., Aral B., Mitze M., Schaffer U., Vaupel P. (1996). Association between tumor hypoxia and malignant progression in advanced cancer of the uterine cervix. Cancer Res..

[8] Bachtiary B., Boutros P.C., Pintilie M., Shi W., Bastianutto C., Li J.H., Schwock J., Zhang W., Penn L.Z., Jurisica I. (2006). Gene expression profiling in cervical cancer: An exploration of intratumor heterogeneity. Clin. Cancer Res..

[9] Davidson S.E., West C.M., Roberts S.A., Hendry J.H., Hunter R.D. (1990). Radiosensitivity testing of primary cervical carcinoma: Evaluation of intra- and inter-tumour heterogeneity. Radiother. Oncol..

[10] Grigsby P.W., Watson M., Powell M.A., Zhang Z., Rader J.S. (2006). Gene expression patterns in advanced human cervical cancer. Int. J. Gynecol. Cancer.

[11] Li X., Huang H., Guan Y., Gong Y., He C.Y., Yi X., Qi M., Chen Z.Y. (2017). Whole-exome sequencing predicted cancer epitope trees of 23 early cervical cancers in Chinese women. Cancer Med..

[12] Kidd E.A., Grigsby P.W. (2008). Intratumoral metabolic heterogeneity of cervical cancer. Clin. Cancer Res..

[13] Ronneberg J.A., Fleischer T., Solvang H.K., Nordgard S.H., Edvardsen H., Potapenko I., Nebdal D., Daviaud C., Gut I., Bukholm I. (2011). Methylation profiling with a panel of cancer related genes: Association with estrogen receptor, TP53 mutation status and expression subtypes in sporadic breast cancer. Mol. Oncol..

[14] Chambwe N., Kormaksson M., Geng H., De S., Michor F., Johnson N.A., Morin R.D., Scott D.W., Godley L.A., Gascoyne R.D. (2014). Variability in DNA methylation defines novel epigenetic subgroups of DLBCL associated with different clinical outcomes. Blood.

[15] Gevaert O., Tibshirani R., Plevritis S.K. (2015). Pancancer analysis of DNA methylation-driven genes using MethylMix. Genome Biol..

[16] Koike F., Satoh J., Miyake S., Yamamoto T., Kawai M., Kikuchi S., Nomura K., Yokoyama K., Ota K., Kanda T. (2003). Microarray analysis identifies interferon beta-regulated genes in multiple sclerosis. J. Neuroimmunol..

[17] Chang Y.E., Laimins L.A. (2000). Microarray analysis identifies interferon-inducible genes and Stat-1 as major transcriptional targets of human papillomavirus type 31. J. Virol..

[18] Duenas-Gonzalez A., Lizano M., Candelaria M., Cetina L., Arce C., Cervera E. (2005). Epigenetics of cervical cancer. An overview and therapeutic perspectives. Mol. Cancer.

[19] Fang J., Zhang H., Jin S. (2014). Epigenetics and cervical cancer: From pathogenesis to therapy. Tumour Biol..

[20] Szalmas A., Konya J. (2009). Epigenetic alterations in cervical carcinogenesis. Semin. Cancer Biol..

[21] Jiao Y., Widschwendter M., Teschendorff A.E. (2014). A systems-level integrative framework for genome-wide DNA methylation and gene expression data identifies differential gene expression modules under epigenetic control. Bioinformatics.

[22] Huang D.W., Sherman B.T., Lempicki R.A. (2009). Systematic and integrative analysis of large gene lists using DAVID bioinformatics resources. Nat. Protoc..

[23] Zheng S.C., Webster A.P., Dong D., Feber A., Graham D.G., Sullivan R., Jevons S., Lovat L.B., Beck S., Widschwendter M. (2018). A novel cell-type deconvolution algorithm reveals substantial contamination by immune cells in saliva, buccal and cervix. Epigenomics.

[24] Uhlen M., Fagerberg L., Hallstrom B.M., Lindskog C., Oksvold P., Mardinoglu A., Sivertsson A., Kampf C., Sjostedt E., Asplund A. (2015). Proteomics. Tissue-based map of the human proteome. Science.

[25] Li X. (2017). Emerging role of mutations in epigenetic regulators including MLL2 derived from The Cancer Genome Atlas for cervical cancer. BMC Cancer.

[26] Vogelstein B., Papadopoulos N., Velculescu V.E., Zhou S., Diaz L.A., Kinzler K.W. (2013). Cancer genome landscapes. Science.

[27] Leonard W.J., O′Shea J.J. (1998). Jaks and STATs: Biological implications. Annu. Rev. Immunol..

[28] Gao Y., Jones A., Fasching P.A., Ruebner M., Beckmann M.W., Widschwendter M., Teschendorff A.E. (2015). The integrative epigenomic-transcriptomic landscape of ER positive breast cancer. Clin. Epigenetics.

[29] The Cancer Genome Atlas Research Network (2017). Integrated genomic and molecular characterization of cervical cancer. Nature.

[30] Burgers W.A., Blanchon L., Pradhan S., de Launoit Y., Kouzarides T., Fuks F. (2007). Viral oncoproteins target the DNA methyltransferases. Oncogene.

[31] Ojesina A.I., Lichtenstein L., Freeman S.S., Pedamallu C.S., Imaz-Rosshandler I., Pugh T.J., Cherniack A.D., Ambrogio L., Cibulskis K., Bertelsen B. (2014). Landscape of genomic alterations in cervical carcinomas. Nature.

[32] Filippova M., Song H., Connolly J.L., Dermody T.S., Duerksen-Hughes P.J. (2002). The human papillomavirus 16 E6 protein binds to tumor necrosis factor (TNF) R1 and protects cells from TNF-induced apoptosis. J. Biol. Chem..

[33] Filippova M., Parkhurst L., Duerksen-Hughes P.J. (2004). The human papillomavirus 16 E6 protein binds to Fas-associated death domain and protects cells from Fas-triggered apoptosis. J. Biol. Chem..

[34] Garnett T.O., Filippova M., Duerksen-Hughes P.J. (2006). Accelerated degradation of FADD and procaspase 8 in cells expressing human papilloma virus 16 E6 impairs TRAIL-mediated apoptosis. Cell Death Differ..

[35] Catani L., Vianelli N., Amabile M., Pattacini L., Valdre L., Fagioli M.E., Poli M., Gugliotta L., Moi P., Marini M.G. (2002). Nuclear factor-erythroid 2 (NF-E2) expression in normal and malignant megakaryocytopoiesis. Leukemia.

[36] Ma X., Zhang J., Liu S., Huang Y., Chen B., Wang D. (2012). Nrf2 knockdown by shRNA inhibits tumor growth and increases efficacy of chemotherapy in cervical cancer. Cancer Chemother. Pharmacol..

[37] Fung F.K., Chan D.W., Liu V.W., Leung T.H., Cheung A.N., Ngan H.Y. (2012). Increased expression of PITX2 transcription factor contributes to ovarian cancer progression. PLoS ONE.

[38] Zhang J.X., Tong Z.T., Yang L., Wang F., Chai H.P., Zhang F., Xie M.R., Zhang A.L., Wu L.M., Hong H. (2013). PITX2: A promising predictive biomarker of patients′ prognosis and chemoradioresistance in esophageal squamous cell carcinoma. Int. J. Cancer.

[39] Imamura T., Kikuchi H., Herraiz M.T., Park D.Y., Mizukami Y., Mino-Kenduson M., Lynch M.P., Rueda B.R., Benita Y., Xavier R.J. (2009). HIF-1alpha and HIF-2alpha have divergent roles in colon cancer. Int. J. Cancer.

[40] Yoshida T., Landhuis E., Dose M., Hazan I., Zhang J., Naito T., Jackson A.F., Wu J., Perotti E.A., Kaufmann C. (2013). Transcriptional regulation of the Ikzf1 locus. Blood.

[41] Noordhuis M.G., Eijsink J.J., Ten Hoor K.A., Roossink F., Hollema H., Arts H.J., Pras E., Maduro J.H., Reyners A.K., de Bock G.H. (2009). Expression of epidermal growth factor receptor (EGFR) and activated EGFR predict poor response to (chemo)radiation and survival in cervical cancer. Clin. Cancer Res..

[42] Choi C.H., Chung J.Y., Kim J.H., Kim B.G., Hewitt S.M. (2016). Expression of fibroblast growth factor receptor family members is associated with prognosis in early stage cervical cancer patients. J. Transl. Med..

[43] Punt S., Houwing-Duistermaat J.J., Schulkens I.A., Thijssen V.L., Osse E.M., de Kroon C.D., Griffioen A.W., Fleuren G.J., Gorter A., Jordanova E.S. (2015). Correlations between immune response and vascularization qRT-PCR gene expression clusters in squamous cervical cancer. Mol. Cancer.

[44] Wilkerson M.D., Hayes D.N. (2010). ConsensusClusterPlus: A class discovery tool with confidence assessments and item tracking. Bioinformatics.

[45] Zhuang J., Widschwendter M., Teschendorff A.E. (2012). A comparison of feature selection and classification methods in DNA methylation studies using the Illumina Infinium platform. BMC Bioinform..

[46] Tusher V.G., Tibshirani R., Chu G. (2001). Significance analysis of microarrays applied to the ionizing radiation response. Proc. Natl. Acad. Sci. USA.

[47] Kent W.J., Sugnet C.W., Furey T.S., Roskin K.M., Pringle T.H., Zahler A.M., Haussler D. (2002). The human genome browser at UCSC. Genome Res..

[48] Ji H., Li X., Wang Q.F., Ning Y. (2013). Differential principal component analysis of ChIP-seq. Proc. Natl. Acad. Sci. USA.

[49] Anders S., Huber W. (2010). Differential expression analysis for sequence count data. Genome Biol..

