# Supplementary material for: Methylation-Based Classification of Cervical Squamous Cell Carcinoma into Two New Subclasses Differing in Immune-Related Gene Expression
Source: Int J Mol Sci. 2018 Nov 15;19(11):3607. doi: 10.3390/ijms19113607 (PMC6275080; doi:10.3390/ijms19113607)
Supplement: Supplementary file 1 [file ijms-19-03607-s001.zip › ijms-374224-SI.pdf]

Int. J. Mol. Sci. 2018, 19, 3607; doi:10.3390/ijms19113607 www.mdpi.com/journal/ijms

International Journal of
Molecular Sciences

Article

Methylation-Based Classification of Cervical Squamous Cell Carcinoma into Two New Subclasses Differing in Immune-Related Gene Expression

Xia Li [1,*] and Yunpeng Cai [1,*]

[1] Research Center for Biomedical Information Technology, Shenzhen Institutes of Advanced Technology, Chinese Academy of Sciences, 1068 Xueyuan Avenue, Shenzhen University Town, Shenzhen 518055, China

* Correspondence: xia.li@siat.ac.cn / bioinflix@126.com (X.L.); Tel.: (86)755-86392203; yp.cai@siat.ac.cn (Y.C.) ; Tel.: (86)755-86392202

Supplementary Figures

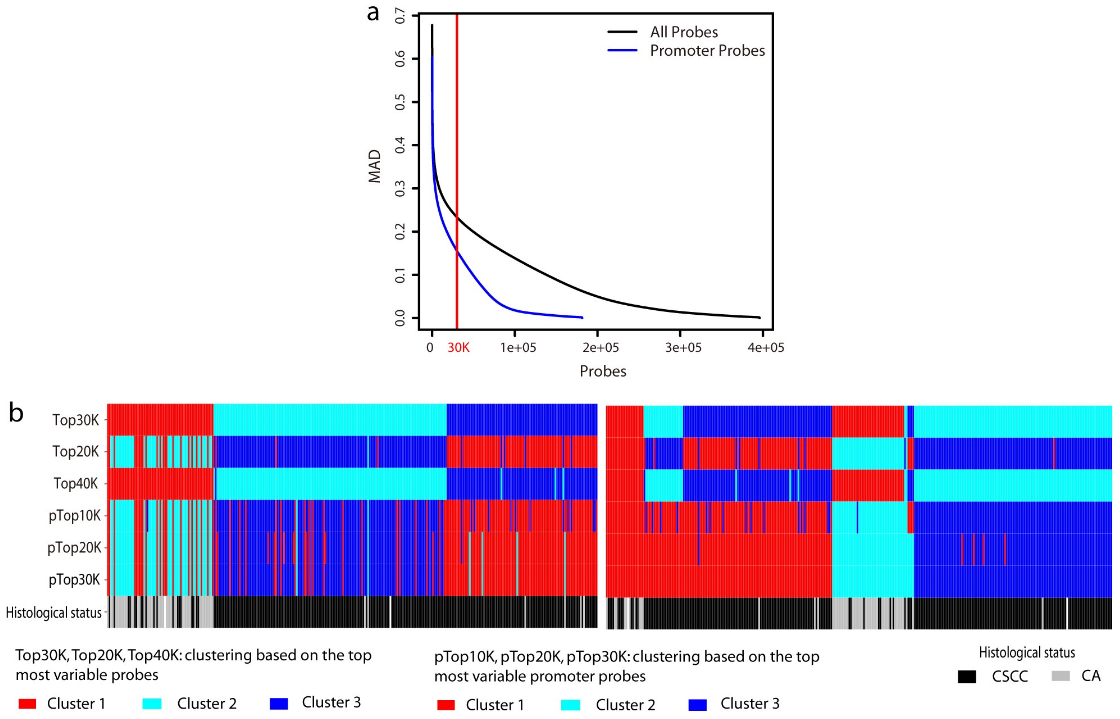

Figure S1. Unsupervised clustering based on the top most variable probes and promoter probes. (**a**) Distribution of MAD values for all probes and promoter probes. Vertical line in red represents the top 30,000 (30K) most variable probes that showed the highest MAD across beta values. We selected these probes for clustering in this study; (**b**) The top 20,000 (Top20K) and 40,000 (Top40K) probes, the top 10,000 (pTop10K), 20,000 (pTop20K), and 30,000 (pTop30K) most variable promoter probes were also used to perform unsupervised hierarchical clustering, respectively. Heatmap shows patients separation in each clustering and their histological status. For clarity, patients were ordered according to their cluster in clustering using the top 30,000 probes (left heatmap), and top 30,000 promoter probes (right heatmap), respectively .

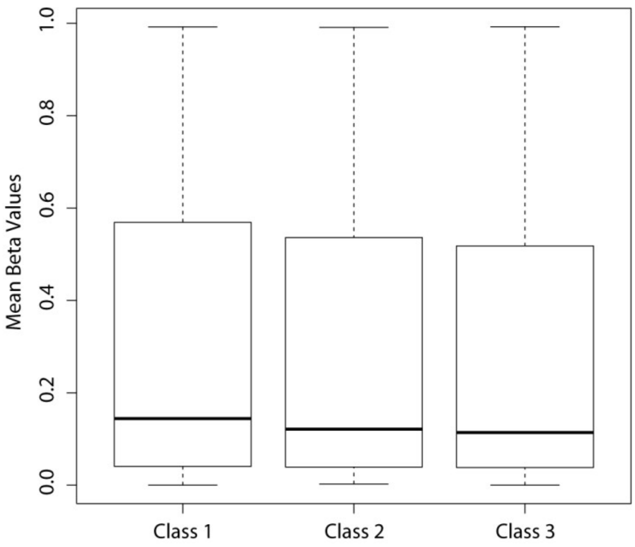

Figure S2. Distribution of mean beta values of probes in each subclass.

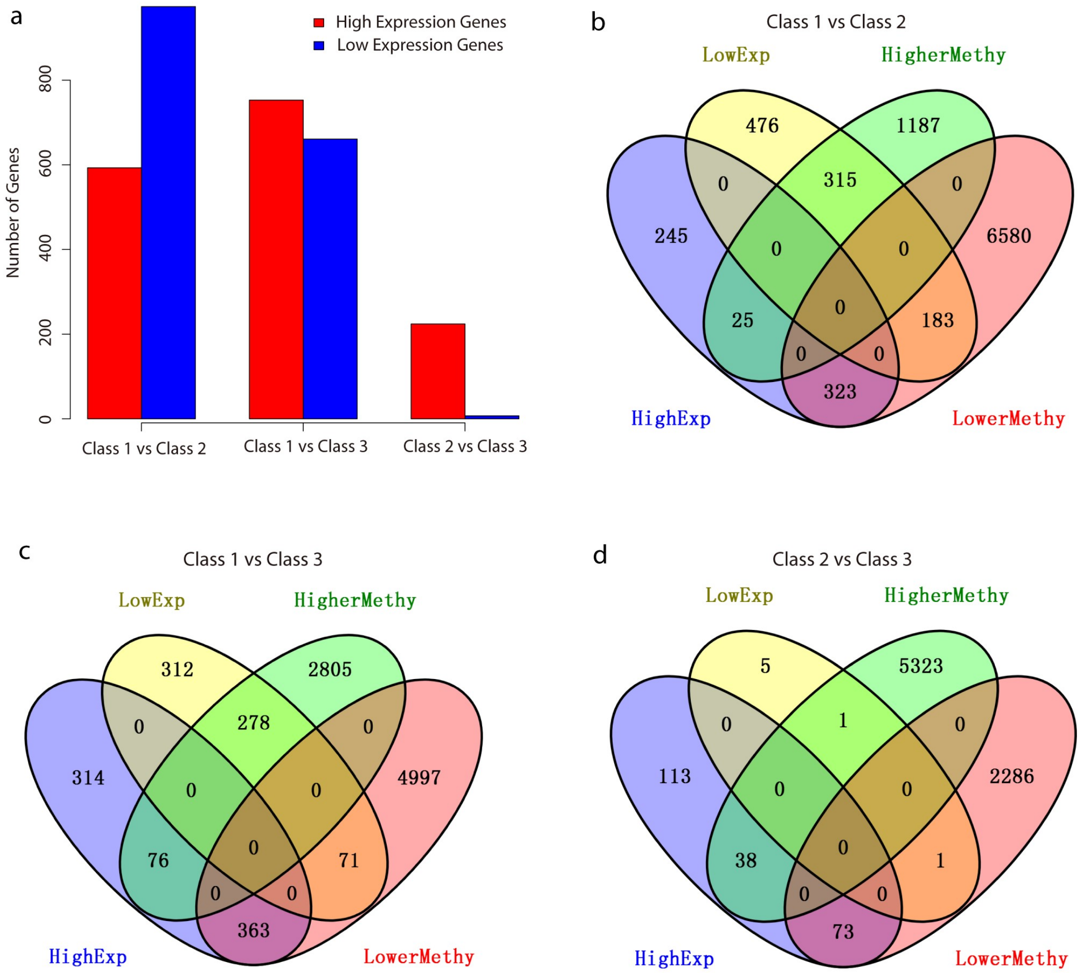

Figure S3. Number of differentially expressed genes and overlap with differentially methylated genes.

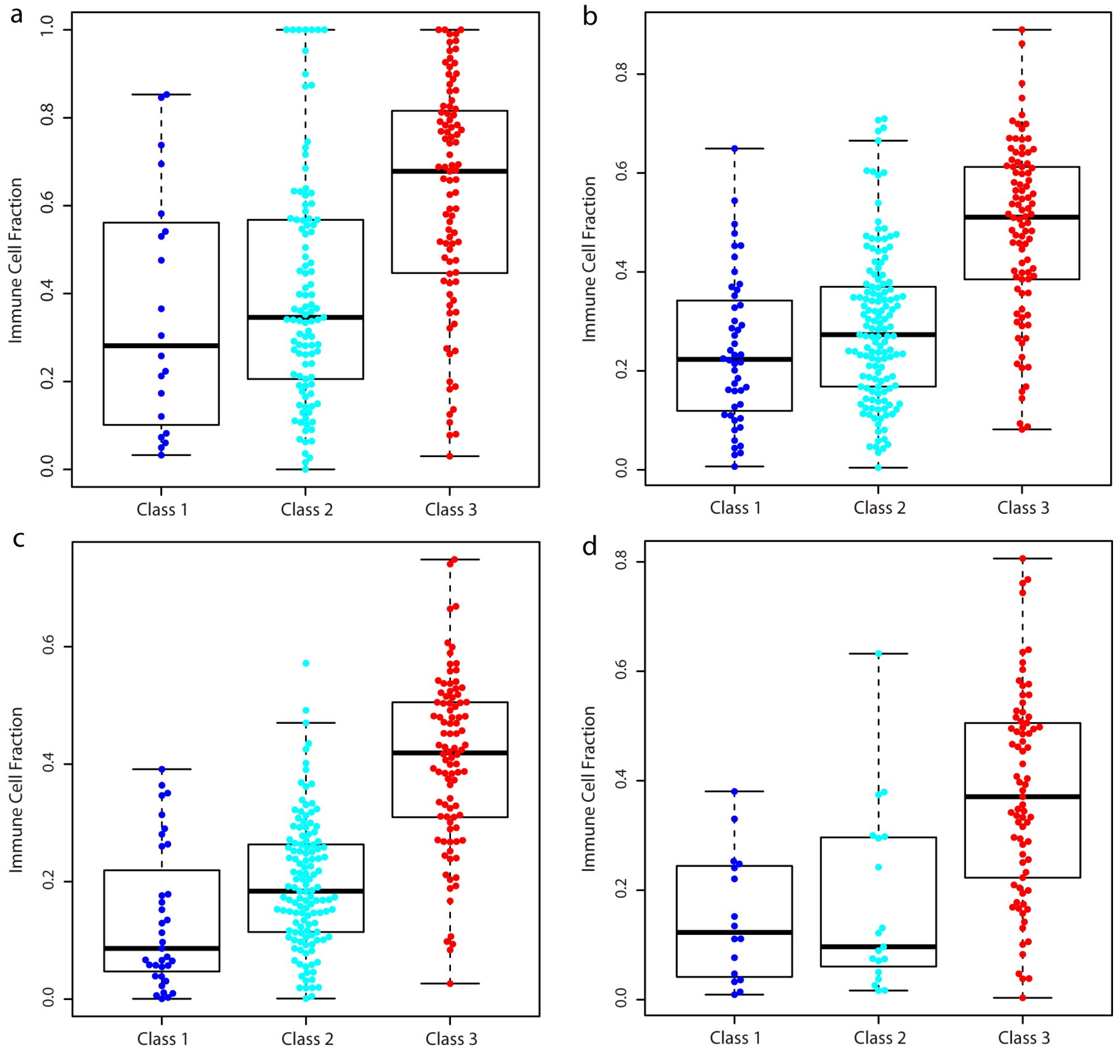

Figure S4. Distribution of immune cell fraction in three groups. Boxplots show the immune cell fraction which was calculated based on top 30,000 most variable probes (**a**), the differentially methylated probes between Class 1 and Class 2 (**b**), Class 1 and Class 3 (**c**), and Class 2 and Class 3 (**d**), respectively.

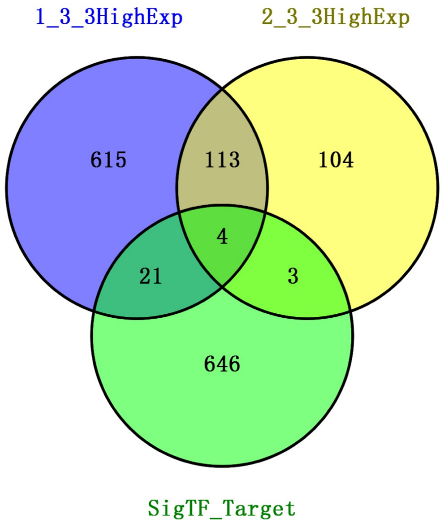

Figure S5. 28 target genes were highly expressed in Class 3. Venn representation of overlaps among highly expressed genes in Class 3 compared to Class 1("1_3_3HighExp"), highly expressed genes in Class 3 compared to Class 2 ("2_3_3HighExp"), and target genes of those 5 significantly enriched TFs ("SigTF_targets").

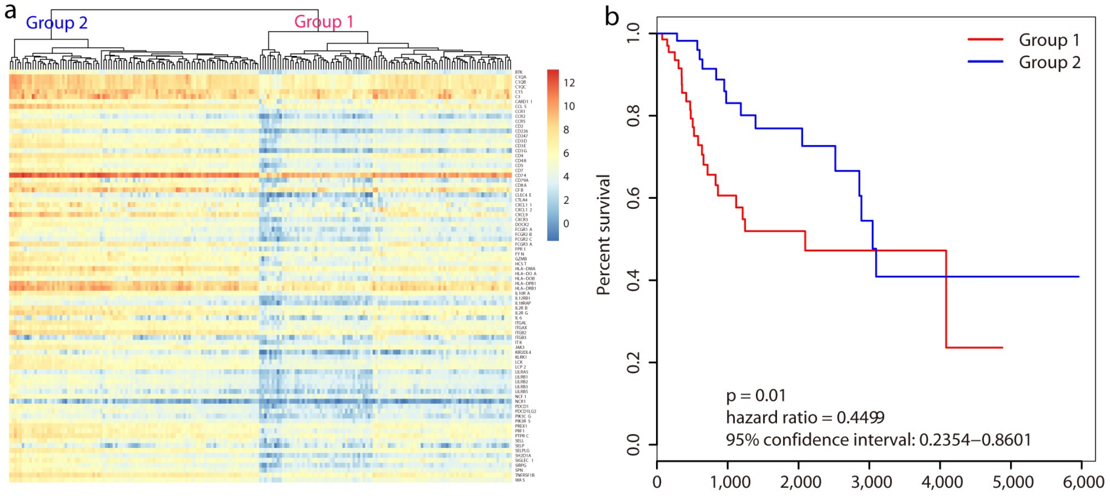

Figure S6. Clinical comparison of the CSCC patients based on the immune genes' expression. We observed a total of 84 immune-related genes were highly expressed in Class 3 as compared with Class 2. The gene expression of these 84 genes could divide patients in CSCC into two groups (**a**). Survival analysis showed the group with high expression displayed better overall survival (**b**).

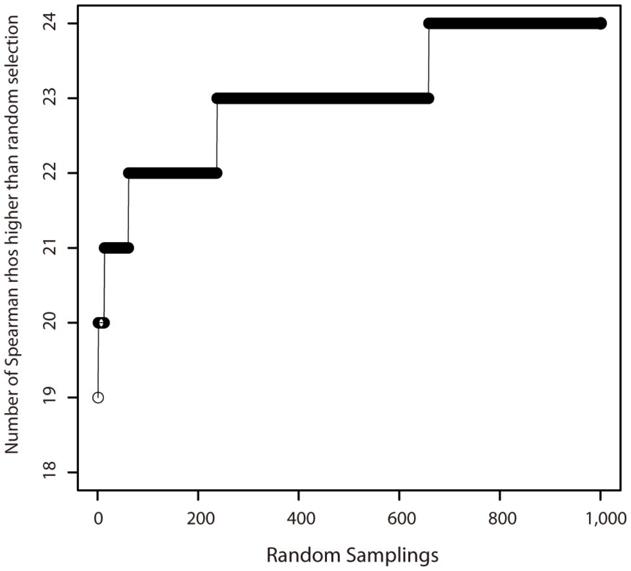

Figure S7. Comparison of Spearman's Rho across 1,000 samplings. Each point represents the number of Spearman's rho among the 24 genes higher than the 24 randomly selected genes' in each sampling.

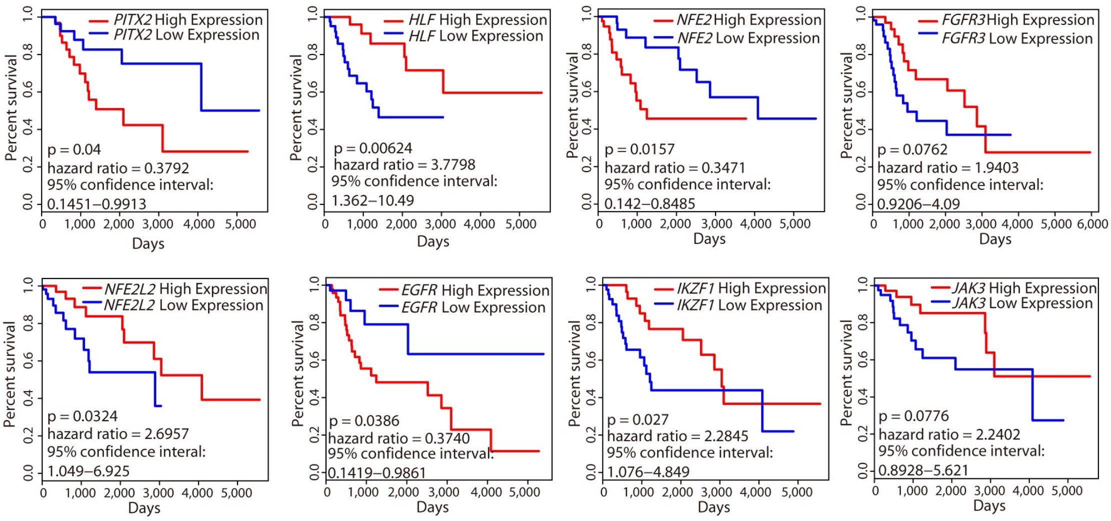

Figure S8. Association between TFs and driver genes' expression and patient survival. Kaplan-Meier curves were constructed to look at the difference in the survival of patients with high and low expression of TFs and driver genes. Patient of high expression was chosen if the expression value was above the fourth quantile, and low expression if below the second quantile. Differences were evaluated by the Log-rank (Mantel-Cox) test.

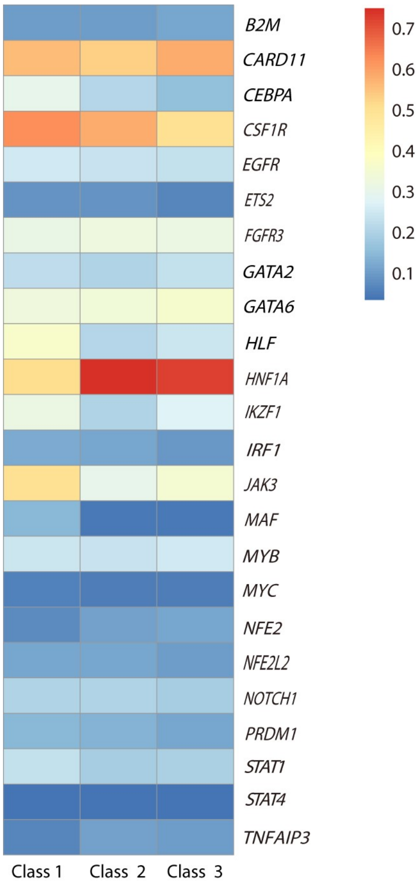

Figure S9. Promoter methylation status of the differentially expressed TFs and driver genes. Heatmap shows the mean beta values of all promoter probes belonging to each gene in each class.

Table S1. Expression of 28 highly expressed TF target genes in cervix and cancer.

Gene	Cervival Cancer Average RPKM[1]	Cervix GTEx RPKM[1]
ARHGAP30	6.1	3
BCL11B	1.9	0.6
CDH13	2.3	12.7
CDK5R1	2.9	0.9
CDKN2B	26.5	6.2
CLIP1	8.1	12.8
FGD2	0.9	3.4
GNAI1	10.8	8.5
HOXA7	1.1	1.3
HOXD10	6.8	12.5
HOXD11	3.7	1.5
HOXD4	0.4	2.3
IRX4	5.4	1.7
LCP1	20.1	4.2
MAFB	18.4	13.7
MARCH1	0.7	1.5
NHLH2	0.3	0.1

PAK6	0.2	1.9
PPP2R3A	3.8	2.8
PVRL1	43.8	7.5
TMEM22	1.1	2.9
C1S	35.2	210.1
CTLA4	2	0.2
SCML4	0.2	0.2
WAS	4.8	2
CARD11	3.3	1.2
CD226	0.2	0.1
FYN	3.5	16.5

[1]Gene expression value was obtained from the Human Protein Atlas.
